# Can cervical disc herniations be provoked *in vitro*?

**DOI:** 10.3389/fbioe.2026.1810527

**Published:** 2026-07-28

**Authors:** Laura Zengerle, Theresa Schilpp, Jan Ulrich Jansen, Deborah Berger, René Jonas, Ann-Kathrin Greiner-Perth, Christian Liebsch, Hans-Joachim Wilke

**Affiliations:** Institute of Orthopaedic Research and Biomechanics, Trauma Research Centre Ulm, Ulm University Medical Centre, Ulm, Germany

**Keywords:** biomechanics, cervical spine, disc herniation, dynamic loading, histology, *in vitro*, novel test method, physiological head movements

## Abstract

**Introduction:**

Neck pain, one of the leading causes of physical disability, can be caused by cervical disc herniation. The purpose of this *in vitro* study was to provoke cervical disc herniations under complex mechanical loading to understand the failure mechanisms and to identify risk factors caused by daily-life activities.

**Methods:**

Six cervical motion segments (3x C4-C5, 3x C6-C7) from four human donors (19–58 years) with Miyazaki degeneration grades II-IV were included. A novel test method was developed to simulate complex neck motions occurring during typical daily-life activities using a dynamic disc loading machine. Long-term effects were simulated by exaggerating and combining these motion patterns. The specimens were subjected to the dynamic loading protocol (n = 3,000 cycles) in the intact state, after 1 mm incision in the posterior longitudinal ligament (PLL), and after complete transection of the PLL. Quasi-static flexibility testing was performed before and after each testing step. For microstructural investigations, histological analysis was performed by cryosectioning three C6-C7 discs.

**Results:**

Using the dynamic loading protocol alone, no herniation could be provoked in intact specimens. Only after the PLL was injured (1 mm incision), a clear prolapse with nucleus extrusion through the defect (n = 1) as well as a protrusion (n = 1) were identified, while complete transection of the PLL did not cause further herniations. Both specimens were less degenerated and from young donors. The extrusion led to a slight increase in range of motion by about 1° in each motion direction. Histological analysis revealed microstructural damage in nucleus pulposus and posterior annulus fibrosus fibers, along with nucleus material flowing through the ruptured annulus in both herniated specimens.

**Discussion:**

Using this novel test method, the simulation of physiological, long-term loading of human cervical discs is feasible. In general, an intact cervical disc does not seem to herniate due to daily-life activities, even under long-term complex motions. The PLL appears to have a main protective effect on the cervical disc, because a herniation only happened when an artificial defect was created. Moreover, the risk of cervical disc herniation might be highest in young patients with low disc degeneration.

## 1 Introduction

Neck pain is categorized as the fourth largest cause of physical disability worldwide (Hoy et al., 2014). Moreover, epidemiological studies have shown that in 36% of all spinal disorders, the cervical spine is affected (Krämer et al., 2014). One common cervical spinal disorder is cervical disc herniation (CDH), which can either be caused by degeneration of disc material, resulting in a soft CDH (Hammer et al., 2016), or the formation of osteophytes, leading to stenosis of the neuroforamina or the spinal canal, the so-called hard CDH (Rohde and Mielke, 2019). The extrusion of disc material often results in narrowing of the nerve root which typically results in symptoms of paralysis around the dermatome. Some studies showed that the C6-C7 motion segments, followed by C5-C6 and C4-C5, are most frequently affected by CDH, with a peak in age between 40 and 50 years (Kelsey et al., 1984; Mundt et al., 1993; Radhakrishnan et al., 1994).

With regard to the development of soft CDH, which, in contrast to hard CDH, can be attributed to successive or sudden failure of annulus fibers and/or endplate cartilage, the exact mechanism behind is not sufficiently understood. Some clinical studies suggest that activities such as lifting loads or frequent driving of heavy vehicles favor soft CDHs (Kelsey et al., 1984; Jensen et al., 1996; Lan et al., 2016). While none of these clinical studies were able to determine the exact movement that caused a CDH, experimental approaches using complex mechanical loading are required to explore the mechanisms underlying soft CDH.

Several *in vitro* protocols have been developed to investigate the mechanisms underlying lumbar disc herniation (Slater et al., 2025). These studies demonstrated that lumbar disc herniation can result from different loading modes, including complex ultimate compressive loading (Adams and Hutton, 1982; Wade et al., 2014; Wade et al., 2015; Shan et al., 2017; Wade et al., 2017), which induces spontaneous structural failures and fractures, as well as cyclic loading (Brown et al., 1957; Adams and Hutton, 1982; Adams and Hutton, 1985; Gordon et al., 1991; Wilke et al., 2013; Wilke et al., 2016; Berger-Roscher et al., 2017; Zengerle et al., 2021; Wade et al., 2022), promoting progressive annular failure. In contrast, research focusing on CDH remains scarce. Previous investigations on porcine cervical intervertebral discs have shown that cyclic loading can also induce herniation in the cervical spine (Tampier et al., 2007). These studies indicate that the herniation process is initiated by the progressive migration of nucleus pulposus material through small annular clefts, leading to accumulation and subsequent delamination within individual lamellae. Moreover, experiments on ovine cervical discs have demonstrated that vibration has a detrimental effect on disc structures and may act as a risk factor for CDH (Gregory and Callaghan, 2011). To the authors’ knowledge, systematic and well-controlled *in vitro* investigations of herniation mechanisms in human cervical intervertebral discs subjected to mechanical loading are currently lacking.

Therefore, the primary objective of this study was to develop and to validate a standardized *in vitro* protocol for inducing cervical disc herniations in human specimens. To accomplish it, this study aimed to identify specific loading conditions and movement patterns that lead to the generation of disc herniations in the cervical spine in order to understand its failure mechanisms. To validate the novel testing protocol, the creation of disc herniations was defined as validation criterion. Furthermore, an analysis of the resulting structural defects was conducted to identify initiating and progressive factors of herniation and to characterize morphological changes associated with cervical disc herniation.

## 2 Materials and methods

### 2.1 Specimens

Six cervical spinal motion segments (3x C4-C5, 3x C6-C7) from four human cadavers (3 male, 1 n.a.) with a median age of 33 years (ranging from 19 to 58 years) were used. The use of human specimens for this *in vitro* study was approved by the ethical committee board of the University of Ulm (No. 35/16). Bony fractures, tumors, and other structural abnormalities were excluded based on CT scans. Disc quality was evaluated using sagittal T1- and T2-weighted and transverse T2-weighted magnetic resonance imaging (MRI) and classified according to the Miyazaki classification (grading from I to V) based on nucleus signal intensity, nucleus structure, distinction of nucleus and annulus, and disc height (Miyazaki et al., 2008). MRIs were also used to verify disc tissue integrity. All included specimens exhibited degeneration grade IV or less (Table 1). The segments were then dissected by removing all fat and muscle tissue while keeping the disc and all functional ligaments intact. The cranial and caudal vertebrae were embedded into PMMA (Technovit 3,040, Haereus Kulzer, Wehrheim, Germany) and flanges were mounted to enable a proper fixation in the testing machines. Prior to dynamic testing, hemi-laminotomy, which was deemed to not significantly affect spinal stability based on previous experimental findings (Montanari et al., 2024), was performed in the posterolateral region of each motion segment to enable observation of the posterior disc with an endoscopic camera.

**TABLE 1:** Human cervical spine specimens from human donors used for this *in vitro* study.

Donor	Segment	Sex	Age in years	Degeneration grade (Miyazaki et al., 2008)
1	C4-C5	m	26	III
​	C6-C7	​	​	II
2	C4-C5	n.a	40	III
3	C4-C5	m	58	IV
​	C6-C7	​	​	IV
4	C6-C7	m	19	II

### 2.2 Novel dynamic loading protocol

The novel loading protocol was developed to induce and characterize the initiation and progression of cervical disc herniations under physiologically realistic conditions. Adapted from a recently established *in vitro* model for the investigation of lumbar disc herniations (Zengerle et al., 2021), the protocol simulates daily motion patterns of the cervical spine that have been associated with CDH development in the literature, including *prolonged forward head bending during excessive smartphone usage* (Rehn et al., 2004; Hansraj, 2014; Cuéllar and Lanman, 2017; Gustafsson et al., 2017). To assess the contribution of excessive motion patterns in additional motion planes, the novel protocol further includes simulation of extension, lateral bending, and axial rotation. These loading conditions were designed to replicate biomechanical loading scenarios of the cervical spine encountered during daily activities, such as sustained *overhead work* and repetitive head rotations common in *assembly-line work*, which may promote CDH.

In order to adapt the existing dynamic loading protocol for lumbar discs to cervical discs regarding axial forces, movement angles, and testing frequencies, and to determine effects of isolated movement components, preliminary experiments using six human specimens (4x C4-C5, 2x C6-C7; 19–40 years; 4 male, 1 female, 1 n.a.) were performed. By means of these preliminary experiments, physiological and superphysiological loading protocols were evaluated to identify conditions potentially capable of inducing prolapse of nucleus pulposus material in the cervical disc without compromising anatomical structures such as ligaments, joint capsules or similar. These experiments demonstrated that moderately increased axial forces in combination with increased ranges of motion lead to prolapse-like bulging of the disc.

**FIGURE 1:**
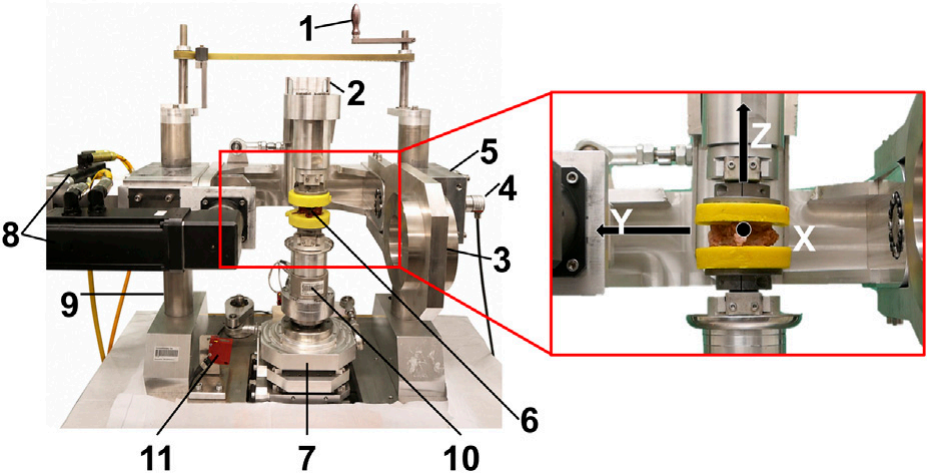
Dynamic disc loading simulator (Wilke et al., 2016) with a mounted cadaveric cervical motion segment used for the induction of intervertebral disc herniation under controlled physiological, superphysiological and maximally complex loading conditions. Components of dynamic disc-loading simulator: *Crank handle (1), swing (2), counterweight (3), angle sensor (4), crosshead with clamping blocks (5), specimen (6), xyz table (7), electric drive (8), column (9), six-component load cell (10), displacement sensor (laser) (11)*. Load directions: + My: flexion, - My: extension, +Mx: left lateral bending, - Mx: right lateral bending, + Mz: left axial rotation, -Mz: right axial rotation, +Fz: compression, -Fz: decompression.

Based on these preliminary findings, all motion patterns in the novel loading protocol were initially simulated within physiological ranges (PHYS, Table 2), derived from *in vivo* kinematic data reported by Anderst et al. (Anderst et al., 2015), adapted to the respective motion segments, and applied using a dynamic disc loading simulator (Wilke et al., 2016; Figure 1). Each activity in a specific motion direction started from a neutral position (*standing head held straight*) and was applied for a total of 100 cycles at 1 Hz, whereas vibration loading was subsequently applied for 1,000 cycles at 3 Hz. Axial loading ranged from 75 N in neutral position (Miura et al., 2002) to a maximum of 100 N for all activities, including vibration (Bernhardt et al., 1999; Bell et al., 2016).

**TABLE 2:** Physiological (PHYS), superphysiological (SUPER), and maximally complex (MAX) loading protocol that was realized by applying the simulated physiological activities (standing, smartphone usage, overhead or assembly-line work, driving buses or heavy trucks) with adequate axial force and physiological range of motion (PHYS), extreme values for single activities (SUPER) or combined version with extreme values in all motion directions (MAX).

​	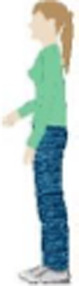	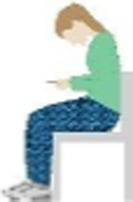	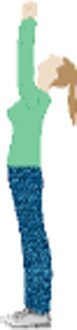	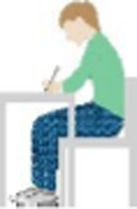	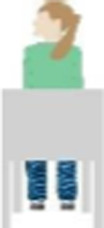	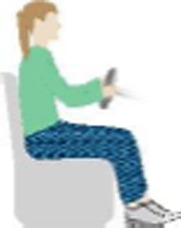
Physiological activity	Standing	Smartphone usage	Overhead work	Assembly-line work	Assembly-line work	Driving buses/trucks
Main motion component	Neutral	Flexion	Extension	Lateral bending	Axial rotation	Vibrations
PHYS: Physiological loading protocol						
Axial force	75 N	100 N	100 N	100 N	100 N	100 N
Frequency	​	1 Hz	1 Hz	1 Hz	1 Hz	3 Hz
Cycles	​	50	50	100	100	1,000
SUPER: Superphysiological loading protocol						
Axial force	75 N	250 N	250 N	250 N	250 N	250 N
Frequency	​	1 Hz	1 Hz	1 Hz	1 Hz	3 Hz
Cycles	​	50	50	100	100	1,000
MAX: Maximally complex loading protocol (i.e., maximally complex motion with combined hyperflexion and hyperextension, lateral bending and axial hyperrotation)						
Axial force	75 N	300 N				
Frequency	​	1 Hz				
Cycles	​	400				

**FIGURE 2:**
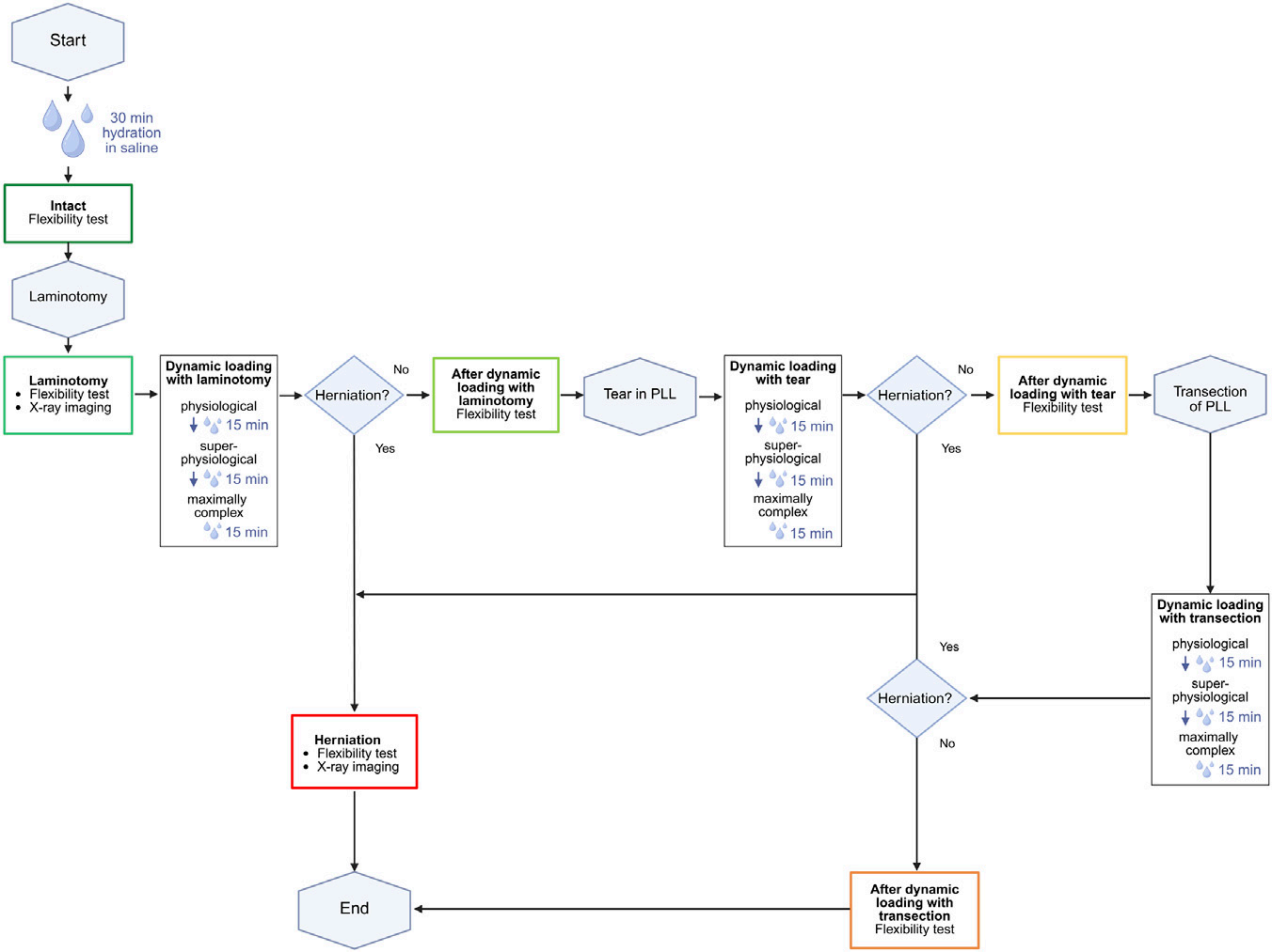
Experimental protocol for inducing intervertebral disc herniation in a cervical functional spinal unit. Colored boxes indicate flexibility assessments performed at different stages of the protocol. Dynamic loading protocols were applied sequentially for physiological, superphysiological, and maximally complex loading conditions. Created in BioRender. Greiner-Perth, A (2026) https://BioRender.com/xnbmg2d.

In a second step, the activities were replicated under superphysiological loading conditions (SUPER, Figure 2; Table 2), corresponding to 125% of physiological values for flexion/extension, 150% for axial rotation, and unmodified lateral bending. Axial loads were increased to 250 N for superphysiological loading.

In a third step, a worst-case loading scenario was applied using a maximally complex motion pattern combining all loading modes simultaneously (MAX, Figure 2; Table 2). This condition was simulated at 1 Hz for 400 cycles with a maximum axial load of 300 N.

### 2.3 Cervical disc hydration capacity

In order to ensure physiologically high hydrostatic pressure in the nucleus pulposus, the specimens were hydrated in physiological saline solution (Fresenius Kabi, Sèvres, France) for 30 min prior to testing and again for 15 min after each dynamic loading step (Pflaster et al., 1997; Zengerle et al., 2021). Generally, the specimens were kept moist throughout the whole experiment (Wilke et al., 1998). The (re-)hydration capacity was determined by measuring the weight of the specimens before and after each hydration period, as well as subsequently after dynamic loading. During the dynamic loading protocol, disc height was continuously monitored using an integrated axial laser measurement system of the dynamic disc loading simulator, with a resolution of 0.01 mm. Changes in disc height were determined before and after each loading protocol (physiological, superphysiological, and maximally complex loading) under an initial axial load of 75 N, which corresponds to the force applied to the disc in standing position (Miura et al., 2002). Disc height changes were evaluated separately for each loading protocol and normalized to the disc height change of the intact state of each functional spinal unit.

### 2.4 Defects

Preliminary experiments indicated that neither an increase in axial compression force alone nor a gradual increase in the range of motion was sufficient to damage the annulus fibers or the posterior longitudinal ligament (PLL) to the extent required to induce a prolapse. The PLL appeared to prevent a soft CDH with extrusion of nucleus material. To overcome this limitation and provoke herniation in the final study, an artificial PLL defect was introduced as part of the experimental model.

**FIGURE 3:**
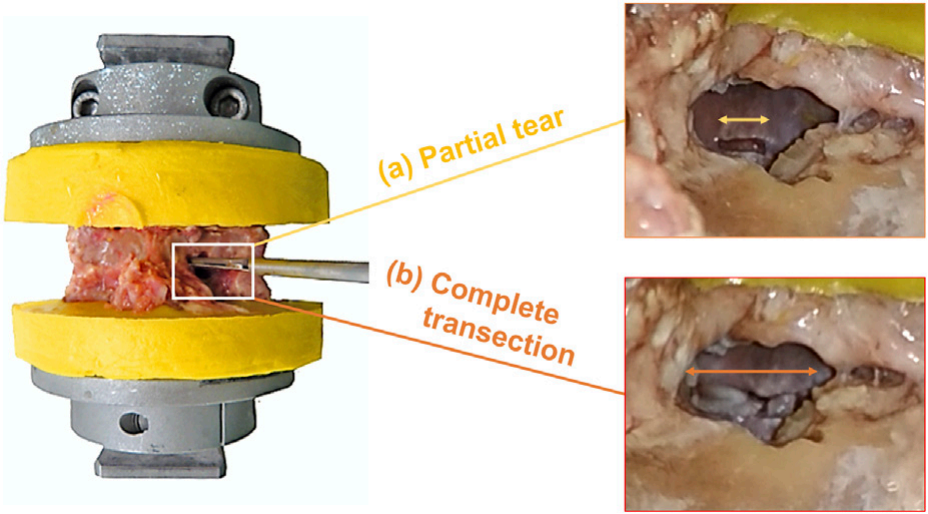
Setting the defects in the posterior longitudinal ligament (PLL): **(a)** tear in the PLL and **(b)** complete transection of the PLL.

Therefore, in specimens where no herniation was induced during the initial dynamic loading session, the PLL was carefully injured by creating a 1 mm incision using a scalpel oriented orthogonally to the ligament fibers (tear defect, Figure 3a). If disc herniation could still not be provoked during the subsequent dynamic loading step, the PLL was completely transected through the pre-existing injury (transection, Figure 3b). In both steps, protection of the posterior annulus fibrosus was given highest priority.

### 2.5 Flexibility testing

The biomechanical properties of the specimens were assessed by performing quasi-static flexibility tests in the intact disc, after laminotomy, after each dynamic loading step, and after a herniation was provoked (Figure 2). After injuring the PLL, no flexibility test was conducted in order to prevent the disc from herniation during quasi-static testing (Zengerle et al., 2021). For determining the flexibility of the specimens, pure moments of ±1.5 Nm were applied in a universal spine loading simulator (Wilke et al., 1994) in flexion/extension, lateral bending, and axial rotation for 3.5 loading cycles (Wilke et al., 1998). Range of motion (ROM) was measured by a motion tracking system (Vicon Nexus 1.4.116, Vicon Motion Systems Ltd., Oxford, United Kingdom) with six cameras (Vicon MX13, Vicon Motion Systems Ltd., Oxford, United Kingdom). The third full loading cycle was used to evaluate flexibility, while the first two cycles were used to minimize viscoelastic behavior and thus to ensure reproducibility (Wilke et al., 1998).

### 2.6 Histological and structural analysis

Histological analysis was performed on three of the six tested cervical discs (3x C6-C7, 19–58 years) based on macroscopic findings following dynamic loading. For sample preparation, all posterior bony elements and transverse processes were resected. The samples were fixed in 3.5% formaldehyde (Otto Fischar GmbH and Co. KG, Saarbrücken, Germany) for 4 days and then rinsed in water for three hours prior to decalcification. Decalcification was performed subsequently for 1 month in a freshly mixed solution of formic acid and tri-sodium citrate buffer (both AppliChem GmbH, Darmstadt, Germany), with final concentrations of 6 mol/L formic acid and 0.4 mol/L tri-sodium citrate, under continuous gentle agitation at 20 cycles per minute (Janke and Kunkel, IKS GmbH, Wetzlar, Germany). The decalcifying medium was replaced every 2 days. Following decalcification, the samples were rinsed in water for three hours.

Each motion segment was sectioned throughout the center of the disc in the sagittal plane and embedded in Tissue-Tek O.C.T. compound (Sakura Finetek Europe B. V., Alphen a. R., Netherlands) followed by deep-freezing under controlled conditions using dry ice at −80 °C for 40 min. Sagittal plane sectioning was performed using a cryotome (Leica CM1950, Nussloch, Germany) at −20 °C. The samples were sectioned from medial to lateral into 60 µm slices. The cryosections were mounted on microscope slides and fixed in an ethanol-based solution (1:1 solution out of 70% ethanol and 100% methanol). Following fixation, sequential cryosections were rinsed with distilled water and coverslipped with Aquatex (Merck KGaA, Darmstadt, Germany).

For structural analysis, sagittal cryosections were examined for defects in intervertebral disc tissue using a bright-field automated stage microscope (Leica DMI 6000B, Leica Microsystems GmbH, Wetzlar, Germany). Mosaic images at five times magnification were generated using the LasX software (Leica Microsystems GmbH, Wetzlar, Germany) and further processed with Adobe Photoshop v27.3.1 (Adobe Inc., San José, CA). Identified defects were classified according to their region within the sagittal disc section (adapted procedure from Wade et al. (2016)), and their frequency was quantified with respect to regional distribution.

### 2.7 Statistics

Data were collected and processed using Microsoft Excel (Microsoft Corp., Redmond, WA, United States). Flexibility data were analyzed using a custom-written MATLAB Script (MathWorks Inc., Natick, United States) to determine the ranges of motion from moment-angle curves for each test step and motion direction. Statistical analysis of differences in spinal flexibility between the test groups consisting of n = 6 specimens was performed using the Friedman test with Bonferroni *post hoc* correction in SPSS27 (IBM Corp., Armonk, NY, United States). The significance level was set to α < 0.05. Grouped data were visualized as medians with value ranges.

## 3 Results

### 3.1 Provocation of cervical disc herniations

**FIGURE 4:**
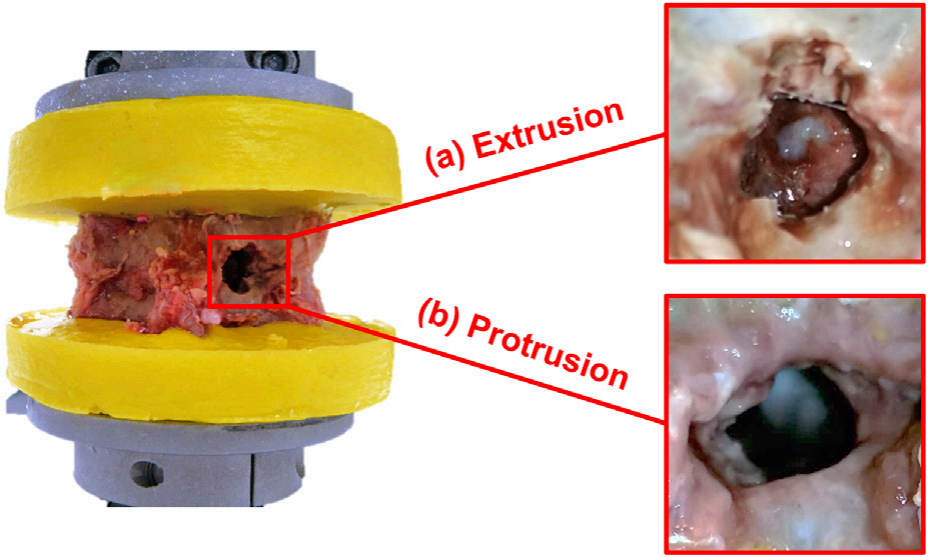
Cervical disc herniation with **(a)** extrusion of the nucleus pulposus (C6-C7, 19 years, m, degeneration grade II) and **(b)** protrusion of the posterior annulus (C6-C7, 26 years, m, degeneration grade II) after setting a tear defect in the PLL, provoked during **(a)** physiological and **(b)** superphysiological dynamic loading, respectively.

In this *in vitro* study, a novel dynamic test method for the examination of cervical disc failure could be developed and was successfully applied to all six specimens. This dynamic test method allowed the simulation of dynamic cervical spine motions representative for daily-life activities across physiological (PHYS) and superphysiological (SUPER) ranges, as well as a maximally complex combination of these motion patterns (MAX). Furthermore, *in vivo* range of motion values could be successfully replicated also for vibrational activities. Using this method, a clearly visible disc herniation with extrusion of nucleus material was provoked under physiological loading in one of the six specimens (C6-C7, 19 years, m, degeneration grade II). In another specimen (C6-C7, 26 years, m, degeneration grade II), a disc protrusion was additionally induced under superphysiological loading. In both cases, herniation occurred only after an artificial tear defect was set into the posterior longitudinal ligament (Figure 4).

In the remaining four specimens, there was no disc prolapse or protrusion, neither in the intact disc nor after damage to the PLL, due to a tear defect or complete transection. Through the posterolateral fenestration, the disc could be observed during testing with the various loading protocols and videos could be recorded. In all six specimens, a clear bulging of the intervertebral disc could be seen during the extension movement with physiological, superphysiological, and maximally complex loading. This was not visible during flexion, lateral bending, axial rotation, and vibration.

### 3.2 Flexibility

**FIGURE 5:**
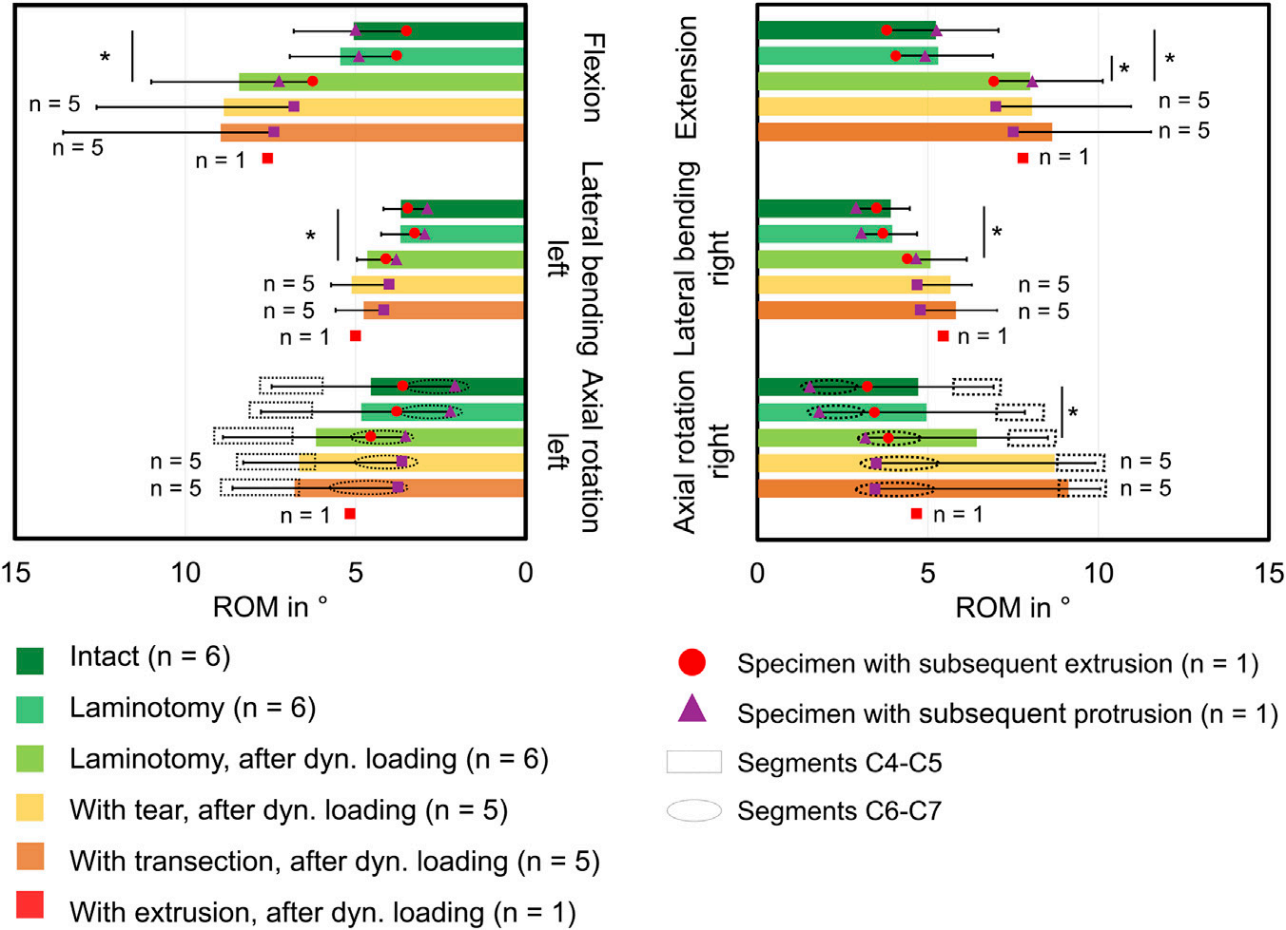
Range of motion (ROM) of ° of the intact cervical disc (green dark shade – intact disc, medium shade – with laminotomy and light shade – after dynamic loading) compared to the condition after setting a tear in the PLL (yellow) and after complete transection of the PLL (orange), each followed by dynamic loading. In addition, ROM of the subsequently herniated disc is shown before extrusion (red dots) and after extrusion (red square), as well as ROM of the subsequently protruded disc before protrusion (purple triangles) and after protrusion (purple squares). The values are described as median and range values. For axial rotation, in which obvious effects of segmental level on ROM were found, C4-C5 ROM is highlighted by rectangular regions and C6-C7 ROM is marked by ellipse regions. *p < 0.05.

In all motion directions, flexibility of the specimens increased with accumulated damage to the posterior structures of the disc and following dynamic loading (Figure 5). Solely in axial rotation, there was an obvious difference in the range of motion between motion segments C4-C5 and C6-C7 (Figure 5). In the one specimen in which a herniation could be provoked following laminotomy and dynamic loading, flexibility increased in all motion directions (Figure 5).

Compared to the intact unloaded disc, dynamic loading significantly increased the range of motion in all directions (p < 0.05) (Figure 5). The median value of flexion increased from 5.1° to 8.4° (p = 0.004). Extension increased from 5.2° in the intact condition to 8° after dynamic loading and laminotomy (p = 0.028), whereas the increase from laminotomy to dynamically loaded with laminotomy was 2.7° (p = 0.028). The median lateral bending to the left increased after dynamic loading by 1° (p = 0.012) and to the right by 1.2° (p = 0.004) compared to the measurement in intact condition. For axial rotation, an increase by 1.7° (p = 0.004) to the right and by 1.3° (p = 0.004) to the left was found between the initial measurement and after the first dynamic loading with laminotomy.

Further damage to the PLL by setting partial and complete tears followed by renewed dynamic loading led to an increased median ROM in all directions of movement, especially in axial rotation to the right.

The extrusion of disc material in one single specimen caused a change in ROM from 6.3° to 7.6° in flexion, from 6.9° to 7.8° in extension, from 4.2° to 5.0° in lateral bending to the right, and 4.4°–5.4° in lateral bending to the left, as well as from 4.5° to 5.2° in axial rotation to the left and from 3.9° to 4.7° in axial rotation to the right.

### 3.3 Disc height

**FIGURE 6:**
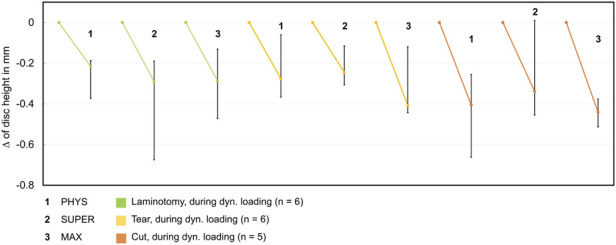
Disc height change of the cervical discs in mm in their different states (green – intact disc, yellow – after setting a tear in the PLL, orange – after complete transection of the PLL) due to dynamic loading with the PHYS – physiological, SUPER – superphysiological, and MAX – maximally complex loading protocol.

Intervertebral disc height decreased with damage to the posterior longitudinal ligament and with increased loading complexity, ranging from physiological to maximally complex loading conditions (Figure 6), as well as over the course of each individual dynamic loading protocol. After placement of the partial tear in the PLL, the median disc height decreased by 0.06 mm. Transecting the PLL led to a decrease in disc height in every measurement. The measurement prior to the physiological loading protocol showed a median decrease of 0.2 mm, of 0.05 mm after physiological loading with PLL transection, and of 0.2 mm after superphysiological loading, each compared to the intact state. The subsequent measurement before maximally complex loading showed a reduction in the median disc height of 0.1 mm.

During the physiological loading protocol itself, a deviation of the disc height was identified during movement in flexion/extension. During lateral bending, axial rotation, and vibration, a change in disc height was observed during both the physiological and the superphysiological loading protocol. During the maximally complex loading protocols, a clear reduction in disc height due to the tear and the complete transection was found (Figure 6). As the disc was hydrated before each measurement, slight increases in disc height were occasionally detected in the barely measurable range in individual cases, similar to observations made during measurements prior to superphysiological loading.

### 3.4 Hydration

**FIGURE 7:**
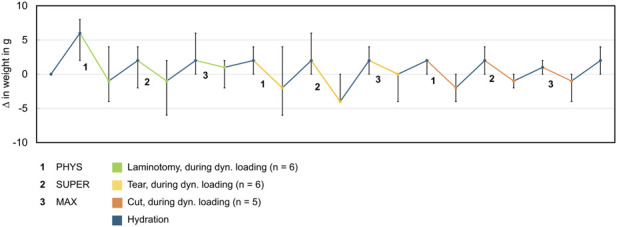
Hydration (blue) and dehydration of the cervical disc in its different states (green – intact disc, yellow – after setting a tear in the PLL, orange – after complete transection of the PLL) followed dynamic loading with the 1 – physiological, 2 – superphysiological and 3 – maximal complex loading protocol, described as change of weight in g.

In general, the weight of the specimens increased after hydration and decreased due to dynamic loading (Figure 7). However, the hydration behavior changed during testing. The highest increase was observed during the first hydration phase before starting the test and during the last hydration after repeated dynamic loading. After almost every dynamic loading protocol, a median weight decrease was documented. After maximally complex loading of the intact disc and after superphysiological loading of the disc with a damaged PLL, no weight loss was observed in the median value compared to the reference value.

### 3.5 Histological and structural analysis

**FIGURE 8:**
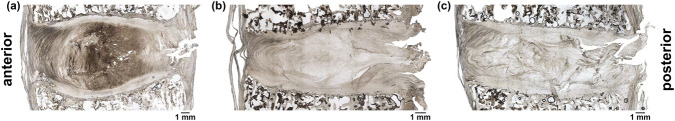
Sagittal cryosections of tested cervical intervertebral discs: Intervertebral disc (C6-C7, 58 years, male, degeneration grade IV) without herniation **(a)**; Intervertebral disc (C6-C7, 19 years, m, degeneration grade II) with nucleus flow and posterior extrusion of nucleus material **(b)**; Intervertebral disc (C6-C7, 26 years, m, degeneration grade II) with posterior nucleus protrusion **(c)**.

The three cervical motion segments (3x C6-C7) selected for structural analysis represented one tested and non-herniated disc as a control (58 years, m, degeneration grade IV), one disc with provoked nucleus protrusion (26 years, m, degeneration grade II), and one disc with provoked nucleus extrusion (19 years, m, degeneration grade II) (Figure 8).

**FIGURE 9:**
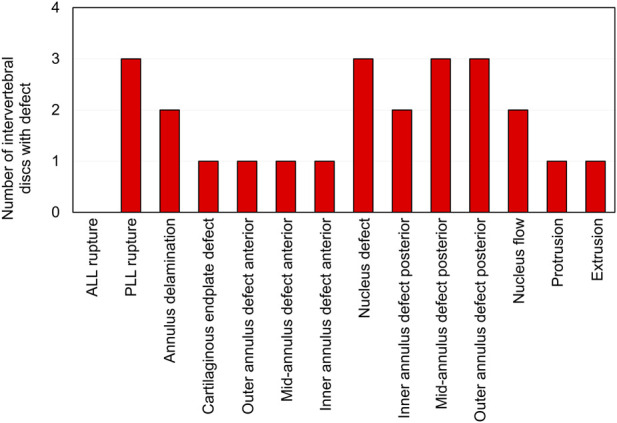
Summary of findings detected by histological and structural analysis of the three histologically evaluated specimens (3x C6-C7).

Structural analysis revealed distinct defect types within the intervertebral disc tissue that were associated with applied dynamic loading. Microstructural defects were classified, and the following categories were identified: Anterior longitudinal ligament (ALL) rupture, defects in the anterior and posterior outer, mid- and inner annulus fibrosus (AF), delamination of annulus fibrosus fibers, defects within the nucleus pulposus (NP), including malposition and nucleus material flow, PLL rupture, and defects of the cartilaginous endplate (Figure 9).

Dynamic loading resulted in a minor defect within the nucleus pulposus without provoked herniation, accompanied by structural damage of the posterior mid- and outer annulus (Figure 8a). The specimen with extrusion exhibited major posterior disruption, pronounced nucleus pulposus malposition, complete delamination of the posterior annulus fibrosus, as well as complete loss of the PLL, indicating that extrusion is associated with severe posterior tissue damage (Figure 8b).

The specimen with protrusion showed intradiscal delamination and posterior annulus defects, with limited nucleus pulposus flow towards the posterior wall, representing an earlier stage of herniation (Figure 8c). Additionally, a posterior defect in the cranial cartilaginous endplate was identified, and an anterior annulus defect was evident. In all specimens, the defect, which had been artificially created in the PLL based on the defect model, was visible in the sagittal cryosection.

## 4 Discussion

### 4.1 Cervical disc herniation

The aim of this work was to develop an *in vitro* protocol for inducing cervical disc herniations (CDH) and to identify the movements and loading conditions that contribute to their occurrence. During the course of the study, in one out of the six specimens, disc herniation with extruded nucleus material could be provoked. One protrusion was generated in another specimen. The novel test method can therefore be considered partially valid.

In both cases, the herniation and the protrusion, a partial tear was set in the PLL prior to the event, as dynamic loading of specimens without defect did not provoke a herniation. These findings suggest that cyclic loading of the intact cervical disc alone does not lead to herniation, neither by physiological nor by superphysiological loads. One reasonable explanation for the occurrence of herniation solely in specimens with an artificial PLL defect is that the intact double-layered PLL exerts a protective effect on the cervical intervertebral disc, thereby reducing the likelihood of cervical disc herniation. This interpretation aligns with the findings of Mercer et al., who demonstrated that the inner nucleus protrudes through the PLL and that the PLL has a main influence in the development of soft CDHs (Mercer and Bogduk, 1999). However, a clinical study by Yamazaki et al., which investigated the trajectory of protruded material in CDH, reported that nucleus pulposus extrusion typically occurred in the posterior median region within the deep layer of the PLL, subsequently followed an oblique course, suggesting that the PLL did not undergo complete structural failure (Yamazaki et al., 2003). While the present experimental protocol, including the defect model for the PLL provides a solid basis for establishing an *in vitro* model of CDH, this approach does not capture the physiological sequence of structural compromise within the cervical intervertebral disc that ultimately leads to herniation, thereby limiting insights into native injury mechanisms. Future studies should aim to enhance the protocol so that herniation development may also be simulated in the absence of an artificially induced defect to better understand injury mechanisms.

Of the two specimens in which herniation could be provoked artificially, only slight degeneration (grade II) (Miyazaki et al., 2008) was observed, whereas all other specimens exhibited more severe degeneration (grades III-IV). This raises the hypothesis that CDH occurs predominantly in intervertebral discs with mild rather than advanced degeneration. The reason for this might lie in lower hydration capacity and more fibrotic tissue of the degenerated cervical disc which shows parallels to the lumbar spine, where disc extrusions only occurred in discs with soft nucleus material (Adams and Hutton, 1985; Zengerle et al., 2021).

Since the requirements for biomechanical investigations of human disc herniations are very high (such as mild degeneration and young donor age (Zengerle et al., 2021)), these specimens are very rare and therefore valuable. However, testing six specimens, which corresponds to the usual number of human specimens used in *in vitro* experiments (Wilke et al., 1998), leads to a certain heterogeneity of tested specimens and segments so that the results have to be interpreted carefully.

### 4.2 Effects on cervical flexibility

Range of motion (ROM) values increased in each specimen over the course of the test. The soft CDH in one specimen, however, did not show any major effects on the ROM compared with the specimens without herniation. Additionally, laminotomy had hardly any influence on the ROM, which is consistent with previous biomechanical findings regarding the contribution of the posterior structures of the cervical disc (Jonas et al., 2020). In contrast, dynamic loading led to a significant increase in the ROM in all motion directions, which might be best explained by released tension due to conditioning of cervical structures, for instance by repeated stretching of the ligaments. Interestingly, any injury to the PLL was found to result in a less destabilizing effect during flexion/extension than during lateral bending and axial rotation, while the injury in the PLL in combination with the right-sided laminotomy could have especially affected the right axial rotation direction. When considering ROM of the segments C4-C5 and C6-C7 separately, a higher degree of flexibility was found in the upper segment, which is consistent with existing data on cervical flexibility (Penning and Wilmink, 1987; Mimura et al., 1989; Wen et al., 1993; Panjabi et al., 2001a; Anderst et al., 2015; Jonas et al., 2018), explaining the large ranges observed in the results. The particularly high range of axial rotation may be explained by variability in the morphology of the uncovertebral joints, favoring head rotations (Tonetti et al., 2005).

Absolute ROM values determined in the present study were overall comparable with previous *in vitro* data on cervical ROM (Wilke et al., 2025), although flexibility tests were conducted under pure moments of 1.5 Nm instead of the 2.5 Nm commonly being recommended for cervical spinal flexibility testing (Wilke et al., 1994; Wilke et al., 1998). The use of lower pure moments was intentionally implemented to minimize the risk of inducing disc herniation already during quasi-static testing. Preliminary tests indicated that the application of lower pure moments prevented the specimens from being further stressed during flexibility testing while still achieving comparable ROM data, especially when using specimens from young donors with low grades of disc degeneration, which generally exhibit higher flexibility (Liebsch et al., 2025). Given that mobility is a key function of the cervical spine and that coupled motions are particularly characteristic of this region (Liebsch and Wilke, 2025), future studies should further advance the understanding of multiparametric flexibility and examine how disc hydration and disc herniation also affect coupled motion patterns.

### 4.3 Hydration capacity of the cervical disc

Hydration analysis showed that intervertebral discs absorb fluid during the water bath and dehydrate under load. Overall hydration capacity of the cervical discs decreased with the course of the experiment. One limitation of the study lies in the shorter rehydration time between the loading cycles, where fluid absorption was less compared to the initial hydration. This behavior is consistent with studies on the hydration behavior of the lumbar intervertebral disc (Pflaster et al., 1997). However, hydration cycles needed to be shortened in order to limit the total time of the experiment during which the specimens remained thawed (Wilke et al., 1998). In order to ensure adequate hydration at the beginning of the experiment, initial hydration was kept for 30 min which was recommended for *in vitro* testing of lumbar discs (Pflaster et al., 1997). A further limitation, however, lies in the limited understanding of the hydration capacity of cervical discs. While for lumbar discs, hydration and rehydration cycles have been described in terms of intradiscal pressure, including diurnal variation under loading and unloading conditions (Wilke et al., 1999), the corresponding behavior of cervical discs is poorly understood. Therefore, it remains unclear whether the level of hydration achieved in this study reflects a physiologically relevant hydration state. Additionally, it is unclear whether a higher degree of hydration could increase the load on disc structures, thereby potentially increasing the herniation risk. Future studies should investigate these aspects in more detail.

In general, the weight measurements used to assess the hydration state of the intervertebral disc should be interpreted in a qualitative manner, as saline solution might not have been absorbed only by the intervertebral disc, but also by soft tissue, and could have been collected in the spinal canal due to the laminotomy. Although weight assessment offers a practical approach of monitoring hydration, it is not the most sensitive measure for quantifying hydration changes within the disc. A more sensitive measure would be intradiscal pressure (Wilke et al., 1999; Schmidt et al., 2016), however, direct measurement of intradiscal pressure in the cervical intervertebral disc was not feasible due to the lack of sensors with sufficiently small diameters for insertion. Future studies should aim to establish protocols incorporating miniaturized pressure sensors for cervical intervertebral discs, enabling more accurate hydration assessment.

Only minor height changes were observed during dynamic loading and following the provocation of a herniation. These limited height changes may be attributed to the distinct anatomical characteristics of cervical intervertebral discs, particularly the presence of uncovertebral joints. Furthermore, these findings could indicate the different functions of the cervical and lumbar intervertebral discs with regard to hydration- and load-related height changes. While the lumbar intervertebral disc tends to have a shock-absorbing effect (McNally and Adams, 1992), the primary function of the cervical intervertebral disc might be to enable adequate flexibility of the cervical spine and thus large head movements, requiring fewer damping effects compared to the lumbar spine. For future biomechanical studies of the cervical disc, it should therefore be critically evaluated, if height measurements add any value.

### 4.4 Application of the novel dynamic loading protocol

A novel dynamic loading protocol simulating typical daily motion patterns of the cervical spine with physiological, superphysiological and maximally complex loading scenarios could be developed. The physiological rotational motions applied are based on previous *in vivo* experiments (Anderst et al., 2015), whereas axial compression forces applied are based on previous *in vitro* measurements (Panjabi et al., 1988; Bernhardt et al., 1999; Patwardhan et al., 2000; Bell et al., 2016; Bell et al., 2019). Whether the loads during the physiological protocol correspond to physiological *in vivo* loads must be critically questioned as it is not known which axial forces occur in each simulated movement. In the test method for investigating lumbar disc herniations, intradiscal pressure was used to calibrate specimen-dependent axial compression (Zengerle et al., 2021). Because of lacking data about intradiscal pressure in the cervical spine *in vivo*, this calibration was not feasible in the current study. Based on limited literature data, it was also not feasible to determine the distribution of forces in the cervical spine across the individual segments (Bernhardt et al., 1999). However, since axial compression was determined in *in vitro* studies using specimens of different ages (Bernhardt et al., 1999; Panjabi et al., 2001b; Miura et al., 2002; Bell et al., 2016), the compression forces applied can be considered an average value. In addition, given that mobility represents the primary function of the cervical spine, load-controlled loading is comparatively less relevant than in the lumbar spine. Accordingly, the application of displacement-controlled, predefined motion patterns represents an appropriate approach for testing cervical motion segments.

Another limitation of the loading protocol is that muscle forces were not simulated, meaning that the applied loading conditions do not fully reflect physiological *in vivo* loads. Incorporating muscle forces would likely increase the loads acting on the disc during the applied motion patterns and could therefore influence the development of disc herniation. Nonetheless, the use of additional superphysiological and maximally complex loading protocols ensured that higher loads, which are potentially more representative of *in vivo* loads, were also simulated.

Vibrations occurring during bus and heavy truck driving have been related to cervical disc herniations (Jensen et al., 1996; Rehn et al., 2004; Lan et al., 2016). Higher frequencies could potentially damage the fibers in the PLL or the posterior annulus. However, the use of higher frequencies in the developed test method was limited by the testing machine. As in preliminary tests, frequencies higher than 3 Hz led to mechanical play and undesirable vibrations in the test machine, a modified test set-up should be used for simulating vibrations with higher frequencies for future studies. Furthermore, the test method has a limitation with respect to the anatomy of cervical motion segments. In preliminary tests, the dynamic loading protocols led to horizontal separation of motion segments with very pronounced uncovertebral joints that extended through the entire intervertebral disc. This *in vitro* study therefore only represents an approximation of physiological *in vivo* behavior during typical daily motion patterns of the cervical spine.

### 4.5 Histological and structural analysis

Histological and microstructural analysis of the three histologically analyzed cervical intervertebral discs revealed distinct patterns of tissue destruction induced by dynamic loading. Among the specimens, one exhibited a nucleus pulposus protrusion and one an extrusion, both of which could be examined in greater detail through cryosectioning. The specimen with protrusion exhibited the most pronounced structural defects, demonstrating that disc failure under complex cervical loading involves posterior nucleus pulposus movement, intradiscal delamination, and progressive destruction of the posterior annulus fibrosus and PLL in protrusion and extrusion.

To provoke herniation under controlled conditions, a defect model was employed based on previous findings that suggested that the cervical PLL has a protective function against disc failure (Mercer and Bogduk, 1999). Therefore, PLL defects were evident in all specimens. In both the protruded and extruded discs, delamination and rupture of the posterior annulus fibrosus fibers were observed. These findings suggest that the integrity of the posterior annulus plays a critical role in the pathogenesis of herniation, potentially more than the cartilaginous endplate. A defect in the cartilaginous endplate was identified only in the one specimen exhibiting protrusion. However, previous histological studies on surgical and autopsy cases by Kokubun et al. indicated that 100% of herniated material comprises cartilaginous endplate tissue and that cartilaginous endplate defects were present in approximately 33% of investigated autopsy cases (Kokubun et al., 1996). These findings suggest that the initial event in cervical disc failure may involve detachment of the endplate from the annulus, highlighting a potential contribution of the endplate in development of herniation. Future studies are therefore required to investigate the structural injury mechanisms of CDH in greater detail, particularly to clarify the currently contradictory findings in the literature.

Across all specimens, defects in the nucleus pulposus were evident as a result of dynamic loading. Flow of nucleus material occurred in both the extruded and protruded cases, illustrating the biomechanical pathway underlying the pathogenesis of disc herniation. Notably, both extruded and protruded discs exhibited only minor degenerative changes, indicating that these discs still had a soft nucleus pulposus. Despite the application of dynamic loading, only minor structural changes were detected in the disc without herniation. This may be attributed to a more advanced degenerative state, characterized by reduced hydration and increased fibrotic changes, which may have limited the tissue’s susceptibility to further mechanical disruption.

## 5 Conclusion

In this study, a novel *in vitro* protocol for the generation of cervical disc herniation was established, enabling the investigation of underlying injury mechanisms of cervical intervertebral discs. Tailored loading protocols incorporating specific motion patterns were developed to replicate physiological and superphysiological loading conditions of the cervical spine and to simulate long-term effects of dynamic loading. In general, an intact cervical disc did not appear to herniate due to daily-life activities, even when being exposed to long-term complex loading. The PLL demonstrated a substantial protective effect on the cervical disc, as herniation could only be successfully induced under dynamic loading scenarios after an artificial PLL defect had been introduced as part of the testing protocol. Furthermore, the risk of CDH may be highest in intervertebral discs with only minor degenerative changes, as herniation was solely generated in discs exhibiting mild degeneration. Nevertheless, the application of the novel testing protocol resulted in one nucleus extrusion as well as one protrusion. Therefore, it can be considered partially valid.

This novel experimental approach provides a promising foundation for future biomechanical research on CDH. Building upon these findings, future studies should expand the protocol to further explore injury mechanisms leading to cervical disc herniation in order to enhance the understanding of herniation initiation and progression and to identify relevant risk factors. Such insights may support the development of improved prevention strategies. Furthermore, the novel *in vitro* model for CDH offers a valuable platform for the investigation, optimization, and evaluation of surgical treatment strategies, including the development and assessment of motion-preserving disc implants.

## Data Availability

The original contributions presented in the study are included in the article/supplementary material, further inquiries can be directed to the corresponding author.
